# 体表经纬穿刺定位法在胸腔镜肺磨玻璃结节手术中的初步应用

**DOI:** 10.3779/j.issn.1009-3419.2020.103.08

**Published:** 2020-08-20

**Authors:** 坤 吕, 永胜 孟, 彤 张, 俊伊 潘, 云靖 李, 长江 冯, 永富 马

**Affiliations:** 1 518116 深圳, 深圳市龙岗中心医院胸外科 Department of Thoracic Surgery, Longgang Central Hospital of Shenzhen City, Shenzhen 518116, China; 2 100101 北京, 中国人民解放军战略支援部队特色医学中心胸外科 Department of Thoracic Surgery, Specialized Medical Center of PLA Strategic Support Force, Beijing 100101, China; 3 100853 北京, 中国人民解放军总医院第一医学中心胸外科 Department of Thoracic Surgery, the First Medical Center of Chinese PLA General Hospital, Beijing 100853, China

**Keywords:** 胸腔镜, 磨玻璃结节, 穿刺定位, Thoracoscopy, Ground-glass nodule, Puncture location

## Abstract

**背景与目的:**

肺磨玻璃结节在胸腔镜手术中如何定位是微创胸外科的重要临床课题, 目前尚无统一的定位方法。本研究拟探讨体表经纬法穿刺定位法在电视胸腔镜肺磨玻璃结节手术中肺结节定位的准确性及安全性。

**方法:**

回顾性分析2018年8月-2019年12月期间我院收治的41例肺磨玻璃结节患者的临床资料, 其中男性28例, 女13例。患者于麻醉后采用体表经纬法穿刺法定位, 然后行胸腔镜下部分肺叶切除术。对手术切除标本测量结节至标记缝线距离和结节至切缘的距离, 统计定位准确率、并发症率和手术切除成功率。

**结果:**

采用体表经纬法穿刺定位法对41例患者共51枚结节进行定位, 准确率达96.1%, 平均定位时间8.3 min。有5例(12.2%)出现穿刺出血, 均胸腔镜下成功止血, 无其他并发症出现。所有患者行胸腔镜部分肺叶切除术, 其中33例行解剖性肺段切除, 8例行肺叶楔形切除术, 手术过程均顺利。测量结节与切缘的距离, 所有标本均 > 2 cm, 达到安全距离, 手术切除成功率100.0%。

**结论:**

在电视胸腔镜肺磨玻璃结节手术中, 体表经纬穿刺定位法是准确、安全、简便的定位方法。

## Abstract (en)

**Background and objective:**

How to locate pulmonary ground-glass nodules in thoracoscopic surgery is an important clinical topic in minimally invasive thoracic surgery. There is no unified localization method at present. This study intends to investigate the accuracy and security of body surface theodolitic puncture localization method in video-assisted thoracoscopic surgery for pulmonary ground-glass nodules.

**Methods:**

The clinical data of 41 patients from August 2018 to December 2019 were analyzed retrospectively, including 28 males and 13 females. After anesthesia, the patient was located by body surface theodolitic puncture, and then partial lobectomy was performed under video-assisted thoracoscopy. The distance from the nodule to the marked suture and the distance from the nodule to the incisal margin were measured, and the accuracy of localization, the rate of complication and the success rate of surgical resection were calculated.

**Results:**

A total of 51 nodules in 41 patients were located by body surface theodolitic puncture localization method. The accuracy rate was 96.1%, and the average location time was 8.3 min. Puncture bleeding occurred in 5 cases (12.2%), all of which were successfully stopped by video-assisted thoracoscopy, and there were no other complications. All patients underwent thoracoscopic partial lobectomy, including 33 cases of anatomical segmentectomy and 8 cases of wedge lobectomy. All the patients in operation process smoothly. The distance between nodule and incisal margin was measured, and all specimens were more than 2 cm, reaching a safe distance. The success rate of surgical resection was 100.0%.

**Conclusion:**

In video-assisted thoracoscopic surgery for ground glass nodules of lung, the body surface theodolitic puncture localization method can be accurate, safe and simple.

随着计算机断层扫描(computed tomography, CT)广泛应用和肺癌筛查普及, 肺磨玻璃结节(ground-glass nodule, GGN)的检出率不断提高^[1]^。其中相当一部分怀疑为恶性的GGN, 需要尽早行外科治疗。电视胸腔镜手术(video-assisted thoracic surgery, VATS)因创伤小, 术后恢复快, 目前已经成为治疗GGN的主要方式^[2, 3]^。很多肺GGN存在位置深、体积小、不易触摸等因素, 外科医生能在术中通过肉眼观察和手指触摸定位结节的成功率较低, 导致定位困难^[4]^。术中无法准确定位肺结节, 会导致手术时间延长, 甚至改变手术方式, 给胸外科微创手术带来了不小的挑战。本研究遵循安全、准确、简便的定位原则, 在VATS术中采用体表经纬穿刺法定位GGN, 取得较好的临床效果, 现报道如下。

## 1 资料和方法

### 1.1 临床资料

将2018年8月-2019年12月中国人民解放军总医院第一医学中心胸外科41例术前计算机断层扫描(computed tomography, CT)发现GGN拟行VATS亚肺叶切除的患者纳入本研究, 其中单发结节34例, 多发结节7例。所有患者术前均无病理诊断, 术前均签署知情同意书。患者临床资料见表 1。

**1:** 患者临床资料(*n*=41) Clinical data of patients (*n*=41)

Variables	Data
Age (Mean±SD, yr)	53.5±9.3
Gender	
Male	28 (68.3%)
Female	13 (31.7%)
Smoking history	21 (51.2%)
Location of nodules	51
Right upper lobe	14 (27.5%)
Right middle lobe	3 (5.9%)
Right lower lobe	13 (25.5%)
Left upper lobe	12 (23.5%)
Left lower lobe	9 (17.6%)
Diameter of nodules (Mean±SD, mm)	9.3±2.5
Pure GGN	23 (45.1%)
Mixed GGN	28 (54.9%)
Distance from nodule to pleura (Mean±SD, mm)	13.3±4.4
GGN: ground-glass nodule.	

### 1.2 纳入标准

① 患者年龄18岁-85岁; ②GGN直径 < 2.0 cm; ③肺结节影像学表现为纯GGN或实性成分≤50%的混合GGN, 可疑恶性病变; ④GGN位于中外2/3肺野。

### 1.3 排除标准

① 肺GGN位于胸膜下, 侵犯脏层胸膜或伴胸膜牵拉; ②肺GGN位于内1/3肺野, 或纵隔胸膜附近, 或结节体表投影点位于肩胛骨区域等不适合经皮穿刺定位者; ③伴有气胸、胸腔积液; ④肺外远处转移; ⑤存在手术禁忌证; ⑥未签署知情同意书。

### 1.4 定位方法

定位于麻醉完成后进行, 患者根据需要采用平卧位或侧卧位。先做体表标记, 确定结节对应于体表的经线和纬线。确定经线：通过CT测量结节垂直于体表的投影点至附近体表标志线(前正中线、后正中线、锁骨中线、腋中线、肩胛线)的距离, 用量尺在体表测量并标记出经线。确定纬线：通过CT确定结节垂直于体表的投影点在相应肋间或肋骨的位置, 在体表标记相应肋间或肋骨画出纬线。经线和纬线相交处即为结节的体表投影点。确定穿刺深度：在CT上沿结节垂直于体表路径, 测量胸壁厚度, 穿刺深度应比胸壁厚度增加2 mm-3 mm, 确保穿刺到肺组织, 同时避免穿刺过深(图 1)。

**1:**
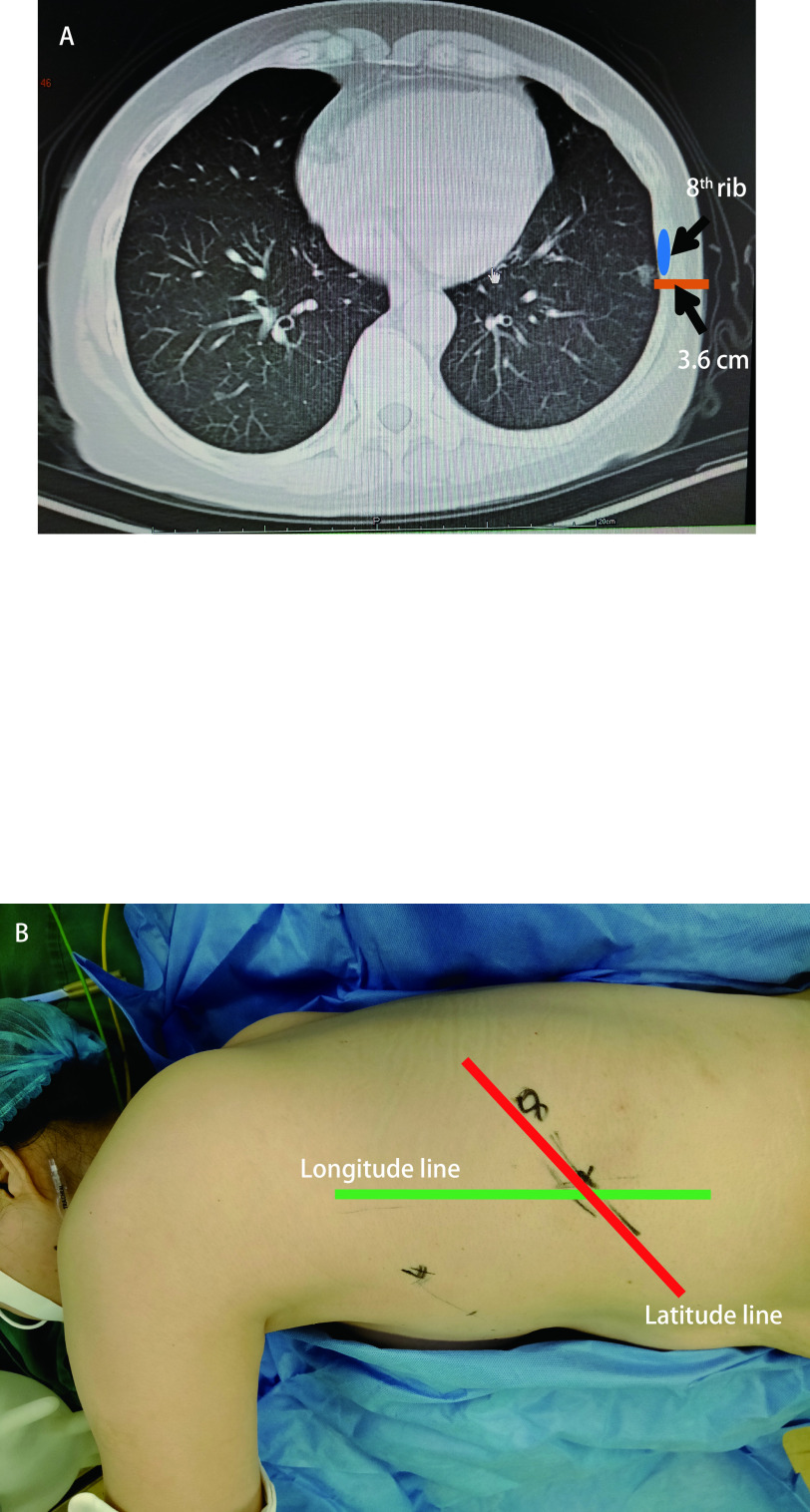
CT测量及体表经纬线确定。A：CT确定经线：侧卧位最高点垂直线；纬线：第8肋骨下缘（避开肋间血管）；穿刺深度：3.6 cm；B：体表划线：经纬线交点即为穿刺点。 CT measurement and body surface longitude and latitude line. A: CT determines the longitude line: vertical line at the highest point in lateral position; latitude line: lower edge of the 8^th^ rib (avoiding intercostal vessels); puncture depth: 3.6 cm; B: Body surface marking: the crossing point of longitude and latitude line is the puncture point. CT: computed tomography.

体表投影点和穿刺深度确定后, 如体表投影点位于肋间(除肋骨下缘), 则直接用22 G穿刺针在体表投影点垂直穿刺达预定穿刺深度即可, 退针后置入胸腔镜, 便可观察到肺表面穿刺点, 即为结节位置, 用缝线打结作为标记。如果体表投影点位于肋骨及肋骨下缘, 无法穿刺, 则在体表投影点附近寻找一处适合穿刺的点, 然后测量穿刺点与结节体表投影点的距离和方向。在穿刺点垂直穿刺达预定穿刺深度, 并在胸腔镜下观察到肺表面穿刺点, 然后根据体表测量穿刺点与结节体表投影点的距离和方向, 在肺表面确定相应位置, 即为结节位置, 用缝线打结作为标记。

### 1.5 手术方法

所有患者在穿刺定位后, 立即消毒铺巾, 行胸腔镜下部分肺叶切除术(解剖性肺段切除或肺叶楔形切除术), 用内镜直切割缝合器切割肺组织时, 肺组织切缘距离标记缝线3 cm以上, 确保足够安全距离。取出肺组织标本后根据标记缝线寻找结节, 确定结节后分别测量结节至标记缝线和切缘的距离, 标本测量后送检病理。

### 1.6 统计学方法

本研究涉及所有数据均采用SPSS 19.0进行分析, 计量资料采用均数±标准差(Mean±SD)表示, 计数资料采用率(%)表示。

## 2 结果

### 2.1 判断标准

肺组织标本切下后, 分别测量结节至标记缝线距离和结节至切缘的距离。结节至标记缝线距离≤1.5 cm为定位成功, > 1.5 cm为定位失败。结节至切缘的距离≥2 cm为手术切除达到安全距离, < 2 cm为手术切除未达到安全距离, 需要扩大切除范围。

### 2.2 定位结果

2018年8月-2019年12月采用体表标记术中穿刺定位GGN患者41例共51枚结节, 平均定位时间为8.3 min。结节至标记缝线距离, 测量结果见表 2, 定位成功49枚结节, 准确率为96.1%。

**2:** 结节至标记缝线距离测量结果 Measurement results of the distance from the nodule to the marked suture

The distance from the nodule to the marked suture (cm)	Nodules
<0.5	14 (27.5%)
0.5-1.0	27 (52.9%)
1.0-1.5	8 (15.7%)
>1.5	2 (3.9%)

### 2.3 定位并发症

共有5例(12.2%)出现穿刺出血, 无其他并发症出现。其中3例患者出现肺组织穿刺点出血, 经胸腔镜下电钩烧灼或缝针成功止血; 2例患者胸壁出血, 经胸腔镜下电钩烧灼后止血。

### 2.4 手术结果

33例行解剖性肺段切除, 8例行肺叶楔形切除术, 手术过程均顺利, 测量结节与切缘的距离, 所有标本均 > 2 cm, 达到安全距离, 手术切除成功率为100.0%。

## 3 讨论

随着人们早期肺癌筛查意识的提高和CT检查的普及, 越来越多的GGN被检出。VATS肺叶或部分肺叶切除术, 是目前GGN的主要治疗方法, 但是许多GGN定位困难, 为VATS手术带来了挑战。

为了解决GGN术中定位的问题, 出现了一系列术前、术中的肺结节辅助定位方法, 包括术前CT引导下经皮穿刺置入Hook-wire、微弹簧圈或注射亚甲蓝、医用胶定位; 术前经电磁导航支气管镜穿刺注射亚甲蓝等染色剂定位; 术中触觉压力感应定位; 术中超声定位等方法, 每种定位方法各有优缺点。术前定位方法会增加患者治疗环节, 其中CT引导下经皮穿刺置入Hook-wire定位较准确, 但有移位脱落的风险, 并有可能造成气胸、胸腔出血等并发症^[5]^; CT引导下经皮穿刺置入微弹簧圈更为安全, 应用较为广泛, 但仍有一定的并发症发生率^[6]^; CT引导下经皮穿刺注射亚甲蓝容易发生染色弥散, 造成穿刺点识别困难^[7]^; 电磁导航支气管镜下穿刺定位较为精准, 但设备及费用昂贵、操作复杂, 短期内难以普及^[8]^。术中定位方法也有不少, 其中通过手指或器械触觉压力感应定位, 简便易行, 但失败率较高, 尤其对于纯GGN^[9]^; 术中超声定位简单、无创, 但是需要肺组织完全塌陷后才能进行, 且依赖于医生的操作经验, 对于深部微小结节和慢性肺病患者失败率高^[4]^; 有研究组采用先体表定位, 术中退出胸腔镜同时鼓肺后穿刺定位, 简便易行, 但是待患肺萎陷后再膨胀, 其与胸壁的相对位置已经发生了移动, 会影响到穿刺定位的准确性^[10]^。

术中解剖标志定位法利用肺结节与胸壁解剖结构相对固定的位置关系, 对照CT图像先大致确定结节所在肺段, 待肺膨胀后在相应肺表面处标记定位。经纬法在前者基础上, 依据肺表面固有的解剖结构所对应的水平或垂直标志线, 在CT图像上确定结节与周围标志线的相对位置关系。肺在萎陷状态下, 可借助结节与各标志线的比例关系来确定结节对应于肺表面的垂直线(经线)和水平线(纬线)进行定位^[11]^。本组研究在经纬法的基础上, 通过CT测量确定肺结节对应于体表的垂直线(经线)和水平线(纬线), 并在胸腔镜置入前进行穿刺, 避免肺移位, 取得了较好结果。本组共定位51例, 经测量结节至标记缝线距离, 49例距离 < 1.5 cm, 定位成功率为96.1%。本定位方法具有以下优势：①安全性高：本方法在麻醉后进行穿刺定位, 穿刺后即刻置入胸腔镜, 如有气胸和出血等情况, 均能第一时间在胸腔镜下给予处理; ②定位准确：在确定体表穿刺点后, 予置入胸腔镜前进行穿刺定位, 此时肺和胸壁的相对位置没有改变, 保证了定位的准确性; 同时不受肺质量的影响; ③流程简单：术前不需要去CT室, 不增加患者治疗环节, 也不需要其他科室医生协助定位; ④操作简便：本方法需要根据CT测量相关距离、体表划线并在胸部穿刺操作, 以上操作对于胸外科医生均不困难, 容易掌握; ⑤无额外费用：本定位方法不需要额外的设备和耗材。

本组有2例, 结节至标记缝线距离 > 1.5 cm, 定位失败, 原因主要是结节位置过深, 导致在确定结节在体表投影的经线和纬线时, 造成角度上的偏差。因此对于靠近肺野中内1/3交界的深部结节, 本方法定位失败率较高。与此相反, 本组8例肺楔形切除, 结节距离肺表面相对表浅, 均准确定位。另外当结节靠近纵隔面, 或结节体表投影点位于肩胛骨、女性乳房等区域, 也不适合用本方法定位。

总之, 对于位置合适的GGN, 体表经纬穿刺定位法是具有安全, 准确, 简便等优点的术中定位方法, 值得推广。但是本研究样本量过小, 仅为初步研究结果, 尚需更大样本研究做进一步验证。
